# Effects of functional decoupling of a leg in a model of stick insect walking incorporating three ipsilateral legs

**DOI:** 10.14814/phy2.13154

**Published:** 2017-02-27

**Authors:** Tibor I. Tóth, Silvia Daun

**Affiliations:** ^1^ Department of Animal PhysiologyInstitute of ZoologyUniversity of CologneCologneGermany; ^2^ Cognitive NeuroscienceInstitute of Neuroscience and Medicine (INM‐3), Research Center JuelichJülichGermany

**Keywords:** Insect locomotion, network model, neuromuscular control

## Abstract

Legged locomotion is a fundamental form of activity of insects during which the legs perform coordinated movements. Sensory signals conveying position, velocity and load of a leg are sent between the thoracic ganglia where the local control networks of the leg muscles are situated. They affect the actual state of the local control networks, hence the stepping of the legs. Sensory coordination in stepping has been intensively studied but important details of its neuronal mechanisms are still unclear. One possibility to tackle this problem is to study what happens to the coordination if a leg is, reversibly or irreversibly, deprived of its normal function. There are numerous behavioral studies on this topic but they could not fully uncover the underlying neuronal mechanisms. Another promising approach to make further progress here can be the use of appropriate models that help elucidate those coordinating mechanisms. We constructed a model of three ipsilateral legs of a stick insect that can mimic coordinated stepping of these legs. We used this model to investigate the possible effects of decoupling a leg. We found that decoupling of the front or the hind leg did not disrupt the coordinated walking of the two remaining legs. However, decoupling of the middle leg yielded mixed results. Both disruption and continuation of coordinated stepping of the front and hind leg occurred. These results agree with the majority of corresponding experimental findings. The model suggests a number of possible mechanisms of decoupling that might bring about the changes.

## Introduction

Legged locomotion is a fundamental form of activity of insects, and in general, of legged animals (Hughes 1952; Wilson 1966; Delcomyn 1981; Orlovsky et al. 1999). Depending on the number of legs, several types of coordination between them have evolved in phylogenesis. In insects, in particular, a number of coordination patterns can be discerned between ipsi as well as contralateral legs during walking (stick insect: Wendler (1966); Graham (1972, 1985); Grabowska et al. (2012); cockroach: Delcomyn (1971); Pearson (1972); Mu and Ritzmann (2008); Drosophila: Wosnitza et al. (2013)). Sensory signals representing load, position, and velocity of a leg segment (e.g., femur or tibia) convey this information to other thoracic ganglia where the local neuronal control networks for the corresponding single leg segments reside. In addition, neuronal signals from the brain also affect the function of the control networks. The weighting of the sensory (peripheral) against the central influences can be and, in fact, is different from species to species. In the stick insect, the peripheral, that is, sensory information dominates the shaping of step movements, hence the coordination patterns (Bässler 1977, 1983; Cruse 1990; Büschges 2005; Borgmann et al. 2007, 2009; Büschges and Gruhn 2007).

In order to understand walking of insects in particular, we need to learn the workings of the neuromuscular systems that bring about intraleg and interleg coordination. In a large number of behavioral and electrophysiological studies (see, among others, the above references) in this field, substantial progress has been made but important details of the coordination mechanisms have remained unknown.

Another way to study this topic is to use modeling techniques in order to uncover the structure and the functional properties of the coordinating mechanisms. Most notably, Cruse and his coworkers have done pioneering work in this field (Cruse 1980; Cruse et al. 1998, 2000; Dürr et al. 2004; Schilling et al. 2007, 2013). Based on different principles, we also constructed a model of the stick insect that could mimic the tetrapod and tripod coordination patterns occurring between three ipsilateral legs during stepping (walking), and moreover, the transition between them (Tóth and Daun‐Gruhn 2016). Admittedly, it is a shortcoming of our model that it comprises three legs only, instead of six. In view of the fact, however, that the interleg coordination between ipsilateral legs was found in the experiments to be much stronger than between contralateral legs (e.g. Borgmann et al. 2009), we think that our present model can still provide useful insights into the workings of the interleg coordination mechanisms of the stick insect during walking. These insights might even be extended to other insects.

An interesting question the answering of which could shed light on the nature of coordination mechanisms is how these mechanisms are changed or impaired if one of the legs is temporarily decoupled from the rest or even becomes defunct. Search movement of the front legs is an example for temporary, reversible decoupling of legs. Loss (amputation) of a leg is, of course, an example for the latter case. There also exist several behavioral studies on the stick insect that use exactly this experimental technique, i.e., amputating or restraining one or two legs, and finding characteristic changes in the walking behavior of these insects. Most notably, Graham (1977) carried out detailed studies of this kind. More recently, Grabowska et al. (2012) also produced important results in this field. In the light of this, it would especially be interesting to find out whether and to what extent simulation results obtained with our model would be in agreement with the experimental findings described in the studies just mentioned. Here, differences between experimental and simulation results, too, could be of importance. Agreement or disagreement of the experimental and simulation results would indicate how accurately the model describes, more precisely, approximates biological reality.

In the simulations to be described below, we specifically decoupled the front, the middle and the hind leg (separately) by using different mechanisms inherent in the model and compared the simulation results with those found in the behavioral studies dealing with this question (Graham 1977; Grabowska et al. 2012). We found a number of important agreements between the experimental and the simulation results. Moreover, it turned out that, in some cases, there were several ways of decoupling a leg. Thus, the model can suggest alternative ways to achieve (nearly) the same results. The physiological suitability and relevance of the possibilities the model suggested will be discussed below.

## Methods

### The model of three ipsilateral legs (3‐leg model)

**Figure 1:**
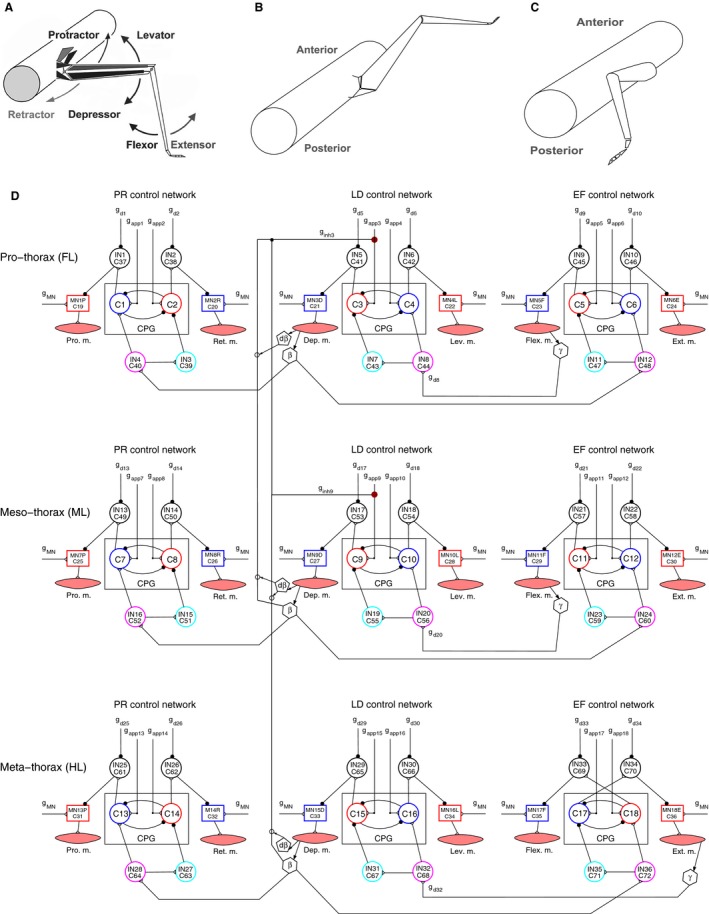
(A) Schematic illustration of a (middle) leg of the stick insect showing three antagonistic muscle pairs that are the most important ones for walking. (Adapted with permission from M.Gruhn unpublished observations.) (B) Lifted, protracted, and extended position of the leg. (C) The leg is on the ground, retracted and flexed. (D) The model of three ipsilateral legs (3‐leg model). Three copies of nearly identical neuro‐muscular networks sitting in the three thoracic segments: the pro‐, meso‐, and metathoracic one, as indicated. They correspond to the front leg (FL), middle leg (ML), and hind leg (HL), respectively. Local networks at one thoracic segment: PR (protractor‐retractor), LD (levator‐depressor), and EF (extensor‐flexor), as indicated, controlling the activity of three antagonistic muscle pairs: m. protractor and retractor coxae (Pro. m. and Ret. m.); m. levator and depressor trochanteris (Lev. m. and Dep. m.); and m. extensor and flexor tibiae (Ext. m. and Flex. m.). CPG in each network: central pattern generator consisting of a pair of mutually inhibitory nonspiking neurons: C1–C2 in the PR, C3–C4 in the LD, and C5–C6 in the EF control network at the prothoracic segment; The arrangement is the same in the two other thoracic segments. *g*_*app*_,_1_, *g*_*app*_,_2_ etc.: (central) input to the CPG neurons (individually variable). MN1P, MN2R etc.: motoneurons driving the corresponding muscles; g_MN_: uniform excitatory input to all motoneurons. IN1‐IN2 etc.: premotor interneurons; *g*_*d1*_, *g*_*d2*_ etc.: (individually variable) inhibitory inputs to these interneurons. IN3‐IN4 etc.: interneurons conveying intrasegmental sensory signals to the corresponding CPG neurons from the other local networks. Hexagons with *β* or *γ* in them: sources of sensory signals encoding position, load, and ground contact. Pentagons with d*β* in them: sources of sensory signals encoding (angular) velocity. The arrows originating at them identify the synaptic pathways they affect. (For a detailed explanation see Tóth and Daun‐Gruhn 2016). Other symbols: empty triangles: excitatory synapses; filled circles: inhibitory synapses. Arrows from muscles to the hexagons symbolize that the sensory signals arise because of mechanical movement due to muscle activity. Intersegmental thick lines connecting the LD systems: inhibitory synaptic pathways from the posterior segment to the next anterior one. *g*_*inh3*_, *g*_*inh9*_: actual synaptic strengths exerting influence on the CPG on which they converge (CPG C3‐C4 and CPG C9‐C10, respectively). These synaptic strengths are completely determined by the actual values of *β* in the next posterior segment. Note that there is no such inhibitory synaptic connection on the LD CPG of the metathoracic (HL) segment.

The first panel of Figure 1 (Fig. 1A) displays the three main antagonistic muscle pairs of a leg of the stick insect. Figure 1B and C depict two characteristic positions of the leg, that is, when the leg is lifted, protracted and extended (Fig. 1B) and when it is on the ground, retracted and flexed. We shall refer to them in the Results. Figure 1D shows our 3‐leg model, which we gradually developed in order to describe and elucidate basic properties of walking, including change of walking direction (Borgmann et al. 2011; Daun‐Gruhn et al. 2011; Tóth et al. 2012, 2013a,b, 2015; Knops et al. 2013; Tóth and Daun‐Gruhn 2016). The model is based on experimental findings (Bässler 1977, 1983, 1993; Graham 1985; Schmitz 1986a,b; Laurent and Burrows 1989a,b; Büschges 1995, 1998; Calabrese 1995; Orlovsky et al. 1999; Schmidt et al. 2001; Ludwar et al. 2005; Hooper et al. 2007; Westmark et al. 2009; Borgmann et al. 2011; Godlewska 2012; Goldammer et al. 2012), and on reasonable physiological assumptions (Daun‐Gruhn et al. 2011; Tóth and Daun‐Gruhn 2011; Tóth et al. 2012; Knops et al. 2013). Each individual local network controls the time evolution of the angle of a specific leg joint. Thus, the protractor‐retractor (PR) network controls the angle *α* between the thorax and coxa, the levator‐depressor (LD) network the angle *β* between the coxa and trochanter, and the extensor‐flexor network the angle *γ*, whose supplementary angle is the angle between the femur and the tibia. The segmental LD control networks play a crucial role in the interleg coordination during walking. The model, in its present form, is capable of reproducing coordination patterns between ipsilateral legs, such as tripod and tetrapod, and the transition between them (Tóth and Daun‐Gruhn 2016). These are characteristic stepping patterns of the legs. During the tripod coordination pattern, the ipsilateral hind and front leg move synchronously having the same phase. They lift‐off and touch‐down alternately with the ipsilateral middle leg. During tetrapod coordination pattern, the three ipsilateral legs lift‐off and touch‐down after each other, that is the order of their lift‐off and touch‐down is hind leg, middle leg and front leg. This is repeated periodically. Examples of both coordination patterns as well as the transition between them are displayed in the Results (Figs 3, 4, 5, 6, 7, 8, 9, 10). Timed temporal inhibition of the anterior LD central pattern generators (CPGs) brings about coordinated lift‐off and touch‐down of the three legs. The mechanism is different for the tripod and tetrapod coordination pattern. It is more complex for the latter coordination pattern. This model was used in the simulations in which we sought to decouple one of the legs from the coordination mechanism of the three legs. (The implementation of the model written in the programming language C is freely available upon request.)

### Ways of decoupling a leg in the model

**Figure 2:**
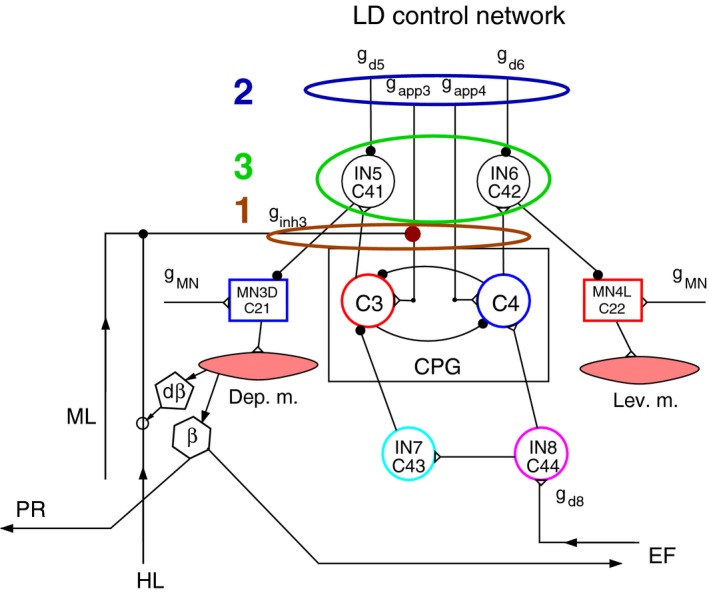
The three basic ways of decoupling one leg from the coordination mechanism of the three legs in the model, exemplified by decoupling the front leg. The numbers 1, 2, and 3 denote these possibilities. 1: decoupling at the intersegmental coordinating synapses from the hind and middle leg. 2: decoupling by changing the (central) drive to the CPG neurons of the LD local network, that is, changing the value of the corresponding conductances g_app_. 3: decoupling by changing the input (conductances *g*_*d5*_, *g*_*d6*_) to the premotor INs in the LD local network. ML, middle leg; HL, hind leg. Other notations are the same as in Figure 1D.

In general, there are three basic ways of decoupling a leg from the coordination mechanism of the three legs in the model. They are schematically illustrated in Figure 2. The first possibility is to disrupt the ‘normal’ function of the synapses responsible for the intersegmental coordination. In this case, their synaptic strength can be set to a permanently high or low value, as required. The central drive (with conductances *g*_*app*_) to the CPGs remains unchanged, as does the (central) input to the premotor interneurons (INs). The second way is to change the (central) drive (the value of *g*_*app*_) to the LD system of the leg in question. The change can, in principle, be an increase or a decrease depending on what steady‐state position the decoupled leg should attain. We shall see that the choice may be different for different legs. Here, the CPGs do not receive their usual input (drive) any more. Finally, the third way is to change the inhibitory input (*g*_*d5*_, *g*_*d6*_ etc.) to the premotor INs of the LD system. The change can again be a decrease or an increase of the inhibition on the particular INs, depending on the desired static position (on the ground or elevated) of the leg. The corresponding CPG is obviously not affected by this procedure. Note that in all of these three cases, the coordination mechanism (cf. Tóth and Daun‐Gruhn 2016) is not destroyed, only some of the elements (e.g., conditions allowing lifting of an anterior leg) have now different parameter values. The result in all cases would, however, have to be decoupling of the desired leg from the full coordination mechanism. The changes of the conductances and drives will be specified in the Results when the simulation results in the individual cases are reported.

## Results

In the experiments, reported in both Graham (1977) and Grabowska et al. (2012), the stick insects walk using both tetrapod and tripod coordination pattern prior to losing one of their legs, or getting it restrained. The former coordination pattern is used mainly by adult animals, or animals walking on a flat surface, the latter by young (first instar) stick insects (Graham 1977), or by ones walking on a steep, declining slope (Grabowska et al. 2012). After amputation of any leg, they almost exclusively used tetrapod or wave‐gait coordination pattern, if any. Thus we, too, treated both cases in the simulations: starting from tripod and starting from tetrapod coordination pattern. As far as the coordination pattern after decoupling (amputation) is concerned, in the simulations, we could not distinguish between wave‐gait and tetrapod coordination patterns, since our model does not include all six legs, that is the contralateral three ones. In the model, all postdecoupling (postamputation) coordinated stepping appears as tetrapod coordination pattern.

There is one more important point to stress. In the experiments, the amputation of a leg is of course irreversible, in the simulations, however, the decoupling of a leg is not. In particular, some parts of the local control network, like intraleg coordination mechanisms of a particular leg remain intact (untouched) in the simulations. It is hard to know to what extent this might hold at a leg amputation. Our results should thus be judged and interpreted in view of this experimental uncertainty.

We report the simulation results concerning the decoupling of each of all three legs, and both starting coordination patterns (tripod and tetrapod). For each leg, all decoupling mechanisms were applied, provided they made sense in the specific circumstances. Comparison to experimental findings was made, and analogy to them was established, where appropriate.

### Decoupling of the front leg

This is a very important physiological case. Grabowska et al. (2012) found that the front legs could temporarily be decoupled in order to carry out search movements (see also Berg et al. 2011), while the two other pairs of legs perform coordinated tetrapod or wave‐gait stepping. It is evident that the decoupling is, in this case, reversible, and is initiated by the animal. First, we deal with the case when tetrapod was the starting coordination pattern (i.e., before the decoupling).

#### Decoupling by perturbing the intersegmental coordinating synapses

**Figure 3:**
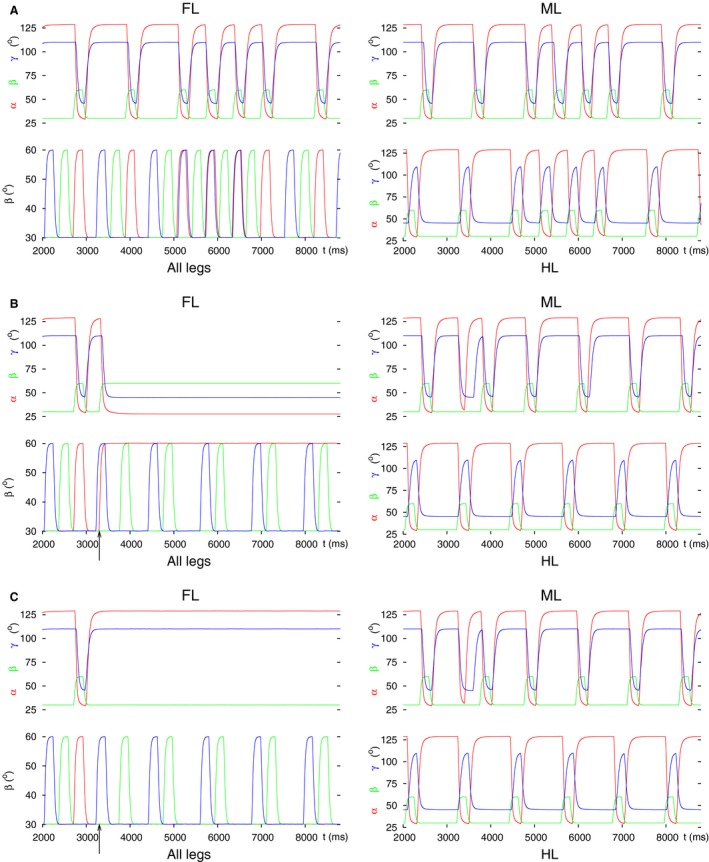
Angular movements of the three legs before and after decoupling of the front leg by perturbing the intersegmental coordination. In all (A, B) and (C) panel FL: angular movements of the front leg as time functions (*α*(t), *β*(t), *γ*(t)) defined in Methods); the ranges for these angles are: *α*: 28° (maximal protraction) – 128° (maximal retraction), *β*: 30° (on the ground) – 60° (maximal elevation), *γ*: 45° (maximal extension) – 110° (maximal flexion); panel all legs: vertical movement of the femur (*β* angles) of the front (red curve), the middle (green curve) and the hind (blue curve) leg; black arrow: start of the decoupling of the front leg; panel ML: angular movements of the middle leg as time functions (in analogy to panel FL), and panel HL: angular movements of the hind leg as time functions (in analogy to panel FL). The two latter panels show the state of the intraleg coordination in the unaffected legs. This also holds for the panels on the right‐hand side in the subsequent figures. (A) Tetrapod and tripod coordination patterns and the transition between them in the 3‐leg model (control case). (B) Decoupling by changing the intersegmental synapse permanently to an excitatory one. (C) Decoupling FL by switching off the intersegmental synapse (setting its conductance permanently to zero). Note the different steady‐state position of the front leg in B (lifted, protracted, and extended, cf. Fig. 1B) and C (on the ground, retracted and flexed, cf. Fig. 1C). Note also the coordinated (alternating) stepping of the middle and the hind leg in these panels after decoupling of the front leg.

First of all, for comparison, we show the tetrapod and tripod coordination patterns, as well as the transition between them in the ‘intact’ 3‐leg model as control (Fig. 3A). To perform the decoupling, we changed the conductance (*g*_*inh3*_) and the reversal potential of the intersegmental synapse on the CPG neuron C3 (Figs 1D and 2), making it excitatory (setting *g*_*inh3*_ to a positive value and the reversal potential to zero), or blocking it (setting *g*_*inh3*_ = 0). The effect of the first type of change is shown in Figure 3B, that of the second in Figure 3C. It is clearly seen that the two types of changes led to different results. In the first case (Fig. 3B), the front leg attained a steady‐state position in which it remained lifted, protracted, and stretched: a good starting position for search movements. In the second case (Fig. 3C), the front leg stayed permanently on the ground, retracted, and flexed, obviously not a good leg position for search movements. Thus, while both changes to the intersegmental synapse on the neuron C3 could stop the front leg's movement, the second type of change did not seem to produce a good steady‐state position of the leg for search movements. It appears therefore that the decoupling of the front leg by this means requires an overall excitatory effect on the CPG neuron C3, a blockade of the synapse (g_*inh3*_ = 0) alone does not suffice. In both cases, however, the coordinated stepping of the hind and middle leg continued, producing what appears to be a tetrapod coordination pattern (bottom left panels in Fig. 3B and C).

As it can be seen in Figure 3, the steady‐state positions of the front leg in Figure 3B and C are complementary. This is a consequence of the intraleg coordination in the model (Fig. 1D). The position signals from the PR and EF neuromuscular systems and the position and load signals from the LD neuromuscular system within a leg bring about the repetitive stepping of the leg in the following manner. If the angle *β* falls below a threshold value, then this means that the leg is approaching ground (ground contact occurs). This initiates the stance phase, hence retraction of the leg. A similar mechanism is at work in the EF system but already at an earlier phase of the stepping period (at a higher threshold value of *β*). Since the front leg remains on the ground, full retraction of the leg will be carried out but after that, no protraction will take place. In normal conditions, flexion occurs in the stance phase, the leg will therefore end up in a flexed position. Clearly, when the front leg lifts off, its movements will be complementary. Hence, the leg will take up a protracted and stretched position, while it stays lifted.

#### Decoupling by changing the drive to the levator‐depressor CPG

**Figure 4:**
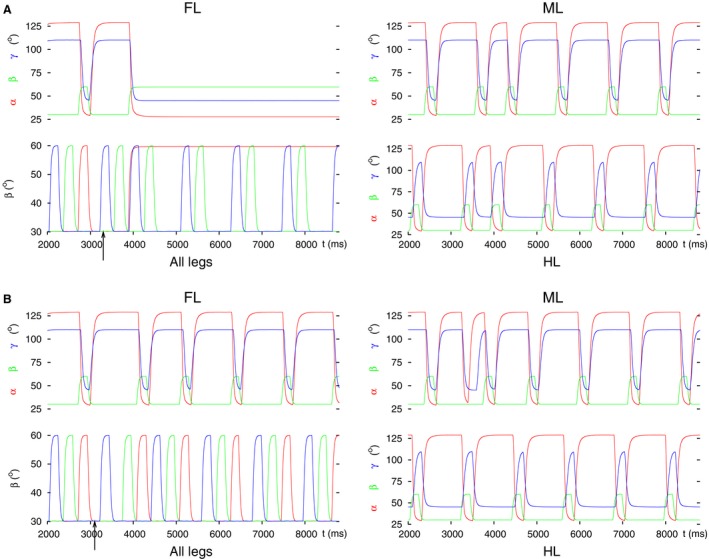
Successful and failed decoupling of the front leg by changing the (central) synaptic drives to the CPG of the LD system of the front leg. Depending on the phase of the stepping period at which the decoupling command is evoked, the decoupling may succeed or fail. (A) successful decoupling. Note the steady‐state position of the front leg. (B) failed decoupling. The same permanent change to the drives (*g*_*app3*_ and *g*_*app4*_) as in A remains ineffective. All notations are the same as in Figure 3.

In the simulations whose results are to be presented here, the input to the levator‐depressor CPG (the conductances *g*_*app3*_ and *g*_*app4*_) was permanently changed. We set a tonic excitatory drive to the levator CPG neuron, and a tonic inhibitory input to the depressor one. We obtained two different kinds of results depending on the timing of the decoupling command. In Figure 4, both types of results are displayed. We found that the decoupling was properly performed if the decoupling command (applying the aforementioned settings) appeared in a time interval which started shortly after lift‐off of the hind leg and lasted until shortly after the lift‐off of the front leg in the unperturbed case, i.e., during normal stepping (Fig. 4A). It was somewhat longer than the half period (≈650 msec with a period of ≈1180 msec). The start of this interval was locked to the lift‐off of the hind leg. No decoupling took place, however, if the decoupling command was evoked in the complementary interval with respect to the stepping period. This interval was, in turn, locked to the lift‐off of the front leg (Fig. 4B). These two intervals alternated, and their lengths added up to the full stepping period. When the decoupling succeeded, the front leg remained lifted, protracted, and stretched as in the previous case (Fig. 3A). At this stage, we can only describe this phenomenon but cannot yet provide a deeper explanation for it because of the high level of complexity of the model. As before, the successful decoupling of the front leg did not destroy the coordinated stepping of the middle and hind leg (Fig. 4A). The front leg here, too, was, in principle, ready to carry out movements (e.g., search movements) independently of the (stepping) movements of the two other legs.

It is worth noting that when we applied both decoupling mechanisms in combination, the results were almost identical to those obtained with changing the synapses of intersegmental coordination, only (see Section Decoupling by perturbing the intersegmental coordinating synapses). This means that the effects of the latter changes dominate those caused by the changes at the LD CPG of the same leg. The reason for this dominance is that the synaptic input to the CPG of the LD system and the intersegmental synapse originating in a posterior segment target the same CPG neuron (e.g., CPG neuron C3 in Fig. 1D). The intersegmental synapse has a much larger conductance than the synapse of the central drive to the CPG. Hence, the activity of the intersegmental synapse determines the output activity of the CPG neuron (Fig. 1D). Since the synaptic activities are periodical, the activity of the intersegmental synapse periodically “overwrites” the central input to the CPG during stepping.

#### Decoupling at the premotor interneurons

**Figure 5:**
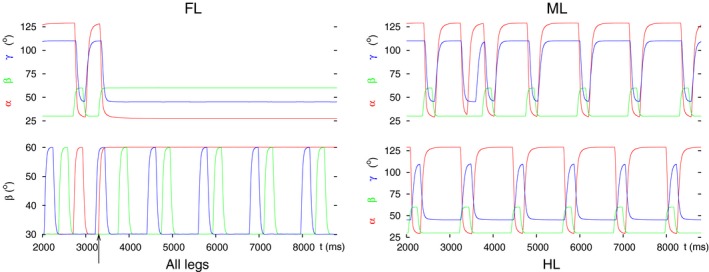
Decoupling of the front leg by inhibiting the inhibitory IN to the levator MN and fully disinhibiting the inhibitory IN to the depressor MN. Note that these changes already sufficed to bring the front leg in the ‘desired’ spatial position: lifted, protracted, stretched. The coordinated stepping of the hind and middle leg continues. All notations are the same as in Figure 3.

The third way of decoupling a leg, in particular the front leg, is changing the input to the premotor INs in the LD control network of the front leg (Fig. 2). The premotor INs control access to the motoneurons (MNs) from the CPGs. It was shown earlier (Tóth et al. 2013a,b) that the premotor INs can completely inhibit the MN activity if they themselves are disinhibited (Fig. 1D). Using this property of the model, the premotor inhibitory IN connecting to the depressor MN of the LD control network was fully disinhibited, suppressing thus the activity of the depressor MN. In addition, the premotor IN to the levator MN was inhibited, hence the activity of this MN was enhanced. The results of these simulations are illustrated in Figure 5. The decoupling command was here, of course, always effective, since the changes to the inputs to the premotor INs directly and permanently affected the activity of the MNs regardless of the actual phase of the stepping period. As it can be seen in Figure 5, the changes of the inputs to the premotor INs proved sufficient for the decoupling of the front leg. Moreover, this leg remained lifted, stretched and protracted. The latter two properties are a consequence of the first one (lifted position), as explained earlier (Decoupling by perturbing the intersegmental coordinating synapses). As in the previous cases, the hind and the middle leg, here too, continued their coordinated stepping using the tetrapod coordination pattern.

We carried out the same kind of simulations in the case, too, when the model started from the tripod coordination pattern. This was done in order to simulate experimental findings which were obtained with young animals (Graham 1977). The results of the first and third variant of decoupling (not illustrated) were in complete analogy to those presented above. We found important differences with variant 2 (changing the input to the LD CPG neurons of the front leg). We could still see both types of results (successful decoupling and continuing stepping of the front leg). The interval within a period where the former occurred, however, became much shorter (about 50 msec, i.e., 8% of the original tripod period ≈615 msec) than in the case with starting tetrapod coordination pattern (about 600 msec, i.e., 50% of the period ≈1180 msec). Therefore, decoupling in this case proved to be very rarely effective, it was practically negligible.

It should be stressed that in all of these simulations, the coordinated stepping of the remaining intact legs: the middle and the hind leg were maintained independently of the starting coordination pattern, in full agreement with the experimental results (Graham 1977; Grabowska et al. 2012). Moreover, the front leg was set free in a spatial position which would make the commencement of search movements easy.

### Decoupling of the hind leg

In behavioral studies, one could often observe that freely walking stick insects suddenly stopped moving their hind legs (in adult animals: M. Grabowska and E. Godlewska, personal communication; in 1st and 2nd instar animals: D. Wetzel and J. Egert, personal communication). They kept them instead in a stretched, retracted position on the ground, whereas the two other legs continued their coordinated stepping. Graham (1977) also studied a similar situation, namely restrained hind leg, in 1st instar animals, albeit the hind leg remained lifted in that case.

This steady‐state spatial position of the hind legs can be regarded as their temporary and reversible decoupling from the two other pairs of legs. In the simulations, we thus strove to reproduce this steady‐state position of the hind leg, while not making any change to the networks that control the two other legs.

Since, according to our 3‐leg model, there are no intersegmental coordinating synapses on the LD CPG of the hind leg, we had here only the aforementioned second and third way of decoupling at our disposal. We start with the second way, that is with decoupling by permanently changing the input to the levator‐depressor CPG. We shall illustrate the results obtained with both ways of decoupling for both starting coordination patterns: tripod or tetrapod.

#### Decoupling by changing the drive to the levator‐depressor CPG

**Figure 6:**
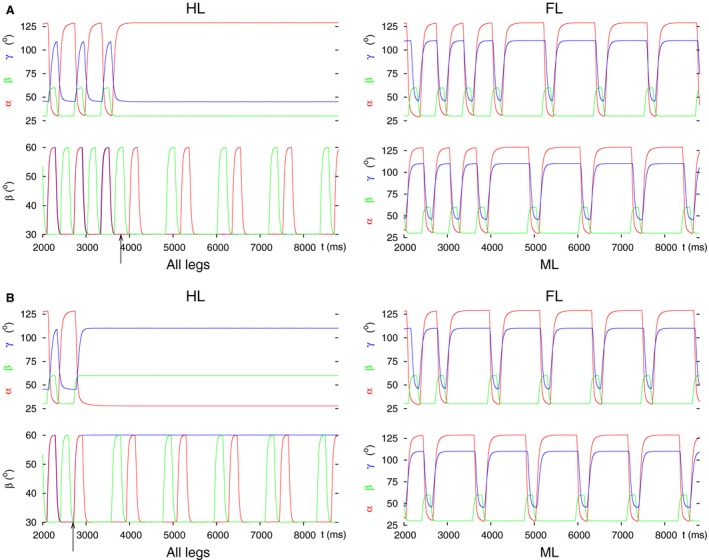
Decoupling of the hind leg by setting the input to both LD CPG neurons to zero. Two types of results emerged: (A) Hind leg on the ground, retracted, and stretched. (B) Hind leg lifted, protracted, flexed. In both cases, the coordinated stepping of the front and middle leg continued. All notations are the same as in Figure 3.

In this set of simulations, we permanently blocked the input both to the depressor CPG neuron and to the levator CPG neuron of the hind leg, that is, we set the conductances *g*_*app15*_ and *g*_*app16*_ to zero. When the starting coordination pattern was tripod, we again obtained two qualitatively different results depending on the phase of the stepping period at which the decoupling command was evoked. These results are illustrated in Figure 6. In one case, the hind leg remained on the ground retracted and stretched, as desired (Fig. 6A). In the case shown in Figure 6B, the hind leg stayed lifted, protracted, and flexed. The two cases occurred periodically, always in the same interval of phases within a stepping period. The interval of the first one (Fig. 6A) was somewhat longer than the complementary interval in which the second case (Fig. 6B) occurred.

The corresponding results are in good agreement with the experimental observations in 1st and 2nd instar animals (D. Wetzel and J. Egert, personal communication), and in essence with those by Graham (1977). In both cases, the coordinated stepping of the front and middle leg continued. This happened despite the fact that, in the ‘intact’ model, the hind leg's CPGs are the origin of the rhythmic activity. This is a remarkable property of the model. That is, even if the hind leg, the original source of the rhythmic activity, fails, the front and middle leg are capable of producing continued coordinated stepping without any external or internal change to them, and, indeed, do so.

With starting tetrapod coordination pattern, however, a strong inhibition of the levator and strong excitation of the depressor CPG neuron was required in order to obtain results similar to those just described, and illustrated in Figure 6. The two groups of results were, however, not identical. The main difference between them was that the results with the hind leg on the ground now appeared in much longer intervals comprising several stepping periods using tetrapod coordination pattern. Thus, this type of decoupling became dominant over the results with the hind leg lifted. Again, the front and middle leg continued their coordinated stepping regardless of the starting coordination pattern.

#### Decoupling at the premotor interneurons

**Figure 7:**
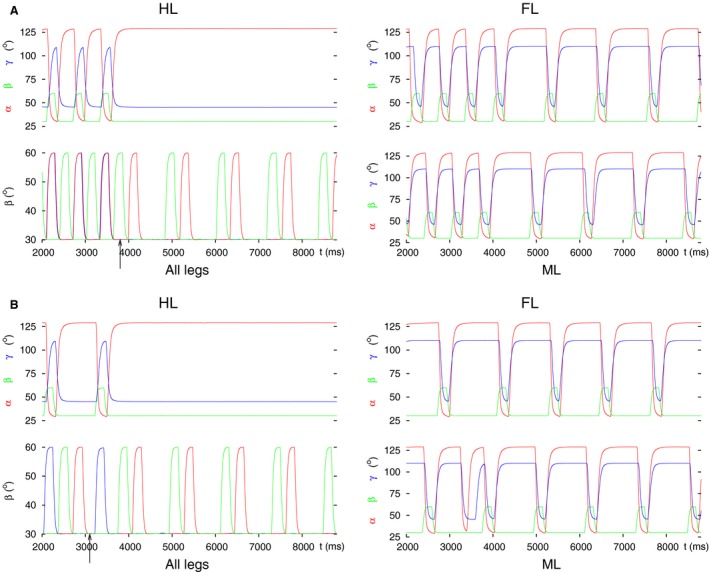
Decoupling of the hind leg by inhibiting the inhibitory IN to the de‐pressor MN and disinhibiting the inhibitory IN to the levator MN. (A) Starting with tripod coordination pattern. (B) Starting with tetrapod coordination pattern. Note that the hind leg behaves the same way in both cases attaining the spatial position seen in the experiments. The coordinated stepping of the front and middle leg continues. All notations are the same as in Figure 3.

In the simulations, we found that disinhibition of the depressor and (strong) inhibition of the levator MN were necessary to decouple the hind leg. These actions proved sufficient, too, for reproducing the experimental findings. This is true irrespective of the starting coordination pattern (tripod or tetrapod) in the simulation. The simulation results are displayed in Figure 7. It shows the hind leg on the ground, retracted, and stretched, as seen in the experiments.

### Decoupling of the middle leg

Decoupling, more precisely, removing (amputating) the middle legs of the stick insect led to profound changes in the intersegmental coordination during walking. Specifically, Grabowska et al. (2012) found that removing the middle legs frequently disrupted the intersegmental coordination between the remaining front and hind legs depending on the terrain on which the animal walked. Thus, it has been of great interest to see whether this could also be reproduced by our 3‐leg model. We did not specify a desired final (static) vertical position of the middle leg in this set of simulations. Thus, the middle leg could be either lifted or on the ground.

Here, we again could apply all three ways of decoupling, since there is an intersegmental coordinating synaptic connection (*g*_*inh9*_) from the meta‐ to the mesothoracic ganglion in the model (Fig. 1D). We shall display simulation results obtained when starting with both tetrapod and tripod coordination pattern.

#### Decoupling by perturbing the intersegmental coordination

**Figure 8:**
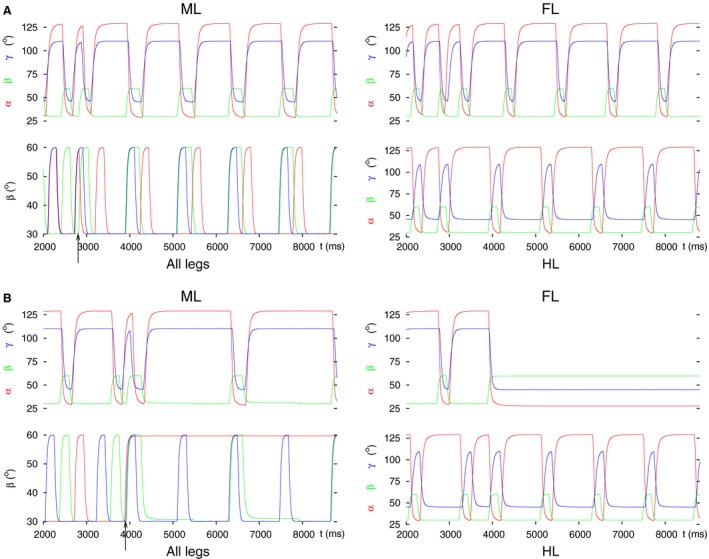
Decoupling of the middle leg at the intersegmental synapse by setting it (with conductance *g*_*inh9*_) permanently excitatory. (A) Starting with tripod coordination pattern. As it can be seen, the middle leg does not stop its rhythmic stepping but that occurs in synchrony with the hind leg's. In addition, the intervals in which both the front leg and the middle leg are lifted partly overlap. An “unusual” coordination pattern is produced. (B) Starting with tetrapod coordination pattern. Here, after the decoupling command (arrow), the middle leg exerts slow rhythmic stepping, the front leg, however, stays permanently lifted, protracted, and stretched. The hind leg continues its normal stepping. In this case, too, the coordination pattern is “unusual”. All notations are the same as in Figure 3.

We permanently changed the intersegmental synapse (with conductance *g*_*inh9*_) to become excitatory (Fig. 1D). The simulation results differ according to the starting coordination pattern. They are illustrated in Figure 8 in which both types of results are displayed (Fig. 8A: tripod coordination pattern, Figure 8B: tetrapod coordination pattern). One can see that when the starting coordination pattern was tripod, the middle leg did not stop stepping but did step in synchrony with the hind leg. In normal tripod coordination pattern, the ipsilateral front and hind leg move synchronously while the middle leg is on the ground. Thus, an unusual coordination pattern was produced by the model that could not be identified either as tripod or as tetrapod one, or as a transition between these two coordination patterns.

When starting with tetrapod coordination pattern, the results were different but the main result remained the same: no physiologically relevant coordination pattern was produced. The details of the failure here were different: the front leg remained permanently lifted, and the middle leg assumed slow stepping activity (of roughly twice the normal period, see the left panels of Figure 8B). The hind leg stepped normally, as in the previous case. Again, we encountered time intervals in which all three ipsilateral legs were lifted (bottom left panel in Figure 8B).

#### Decoupling by changing the drive to the levator‐depressor CPG

**Figure 9:**
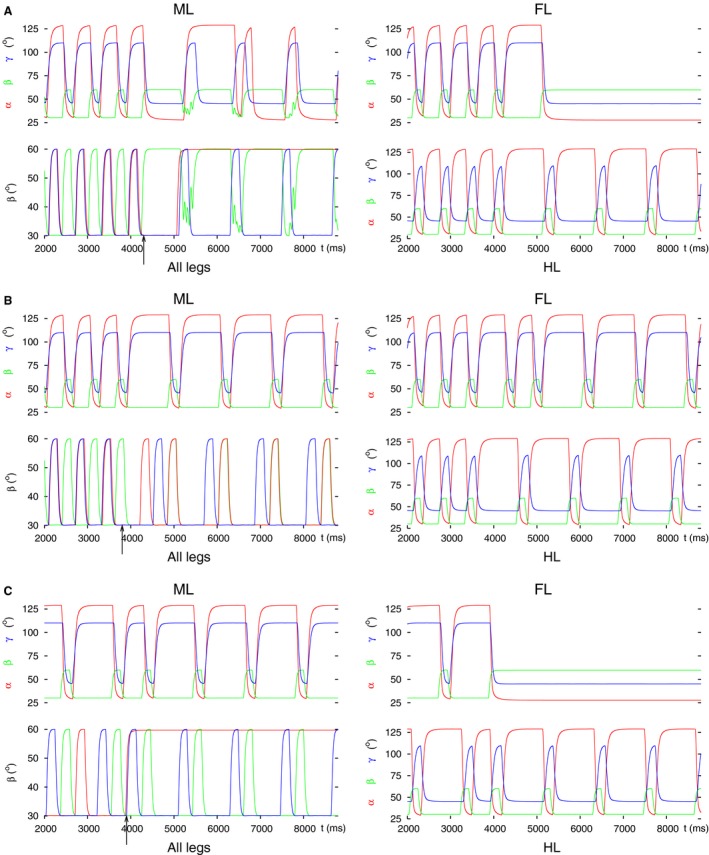
Decoupling of the middle leg by permanently changing the input to its LD CPG. Three typical results are illustrated. The first result was obtained by applying (strong) excitatory input to the levator and (strong) inhibitory input to the depressor CPG neuron. The second and third one arose, when there was no input to the CPG (both input conductances were set to zero; Figure 1D). (A) The middle leg does not have proper ground contact; the front leg remains lifted, protracted, and stretched; the hind leg continues its normal stepping. This result was obtained with both starting coordination patterns (tripod, tetrapod). The result displayed is with the starting coordination pattern tripod. (B) All three legs continue stepping but the front and middle leg step in synchrony. This result could only be seen with starting tripod coordination pattern. (C) The middle and the hind leg continue tetrapod stepping but the front leg remains permanently lifted (protracted and stretched). This result clearly shows a failed decoupling of the middle leg but a successful decoupling of the front leg. It occurred only with starting tetrapod coordination pattern. All notations are the same as in Figure 3.

Next, we permanently changed the input to the LD CPG of the middle leg in a similar manner as we did with the other legs. Thus, we applied strong excitation to the levator CPG neuron and strong inhibition to the depressor CPG neuron. This resulted in the stepping behavior shown in Figure 9A.

Here, the middle leg still continued with rhythmic movements but had no proper ground contact. Moreover, the front leg remained permanently lifted (protracted and stretched). The coordinated stepping therefore broke down. This result appeared with both starting coordination patterns (tripod and tetrapod). The result illustrated in Figure 9B could only be obtained when there was no input to the CPG (*g*_*app9*_ and *g*_*app10*_ were set to zero) and the simulation started with the tripod coordination pattern. In this case, all three legs continued stepping after the decoupling command but the front and middle leg stepped in complete synchrony. This is contrary to what happens in the tripod or tetrapod coordination patterns. In the former, the hind and front leg step together, while in the latter, the hind, middle and front leg step after each other. Obviously, this stepping pattern does not constitute a transition between the tripod and tetrapod coordination patterns, either.

A third type of results appeared at the same (zero) values of the input conductances to the CPG, and with starting tetrapod coordination pattern, only (Fig. 9C). Here, the decoupling of the middle leg clearly failed, since the hind and the middle leg continued their coordinated stepping after the decoupling command. However, the front leg appeared to be properly decoupled. This is somewhat surprising but a detailed quantitative analysis of this specific result would go beyond the objectives of this paper.

We also note that when the two previously described ways of decoupling were used in combination, decoupling the middle leg via the intersegmental synapse dominated the outcome of the simulations. That is, we obtained, in essence, the same results irrespective of whether changes to the input to the CPG were applied.

#### Decoupling at the premotor interneurons

**Figure 10:**
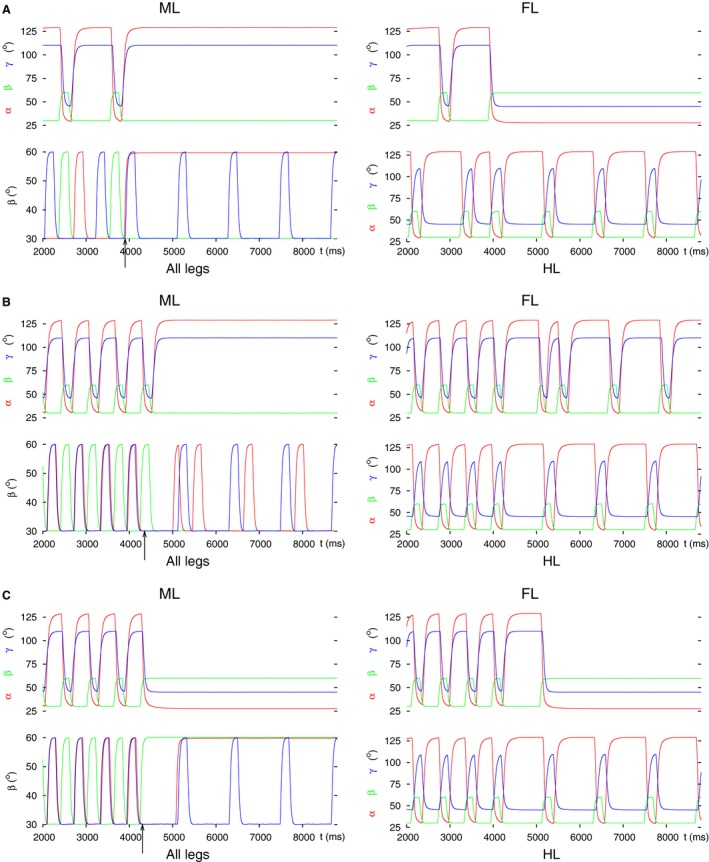
Decoupling of the middle leg via premotor inhibitory INs. (A) Premotor IN to the depressor MN is inhibited, while the one to the levator MN is disinhibited. As a result, the middle leg permanently remains on the ground (retracted, flexed). The front leg stays lifted (protracted and stretched), while the hind leg continues normal stepping. (B) The inputs to the premotor INs are the same as in A but the decoupling command arrives at a different phase of the stepping period. As a result, the front leg and the hind leg now perform coordinated stepping. (C) Premotor IN to the depressor MN is disinhibited, while the one to the levator MN is inhibited. The middle leg therefore stays lifted (protracted and stretched). The front leg also stays lifted as in A. Here, the starting coordination pattern in A was tetrapod, while in B and C tripod. All notations are the same as in Figure 3.

Finally, we used the premotor inhibitory INs to decouple the middle leg from the system of coordinated stepping. When we inhibited the depressor and disinhibited the levator MN, the middle leg stayed permanently lifted. Exchanging inhibition and disinhibition, the middle leg remained on the ground. In both conditions (steady‐state position of the middle leg), two different types of behavior emerged. They are illustrated in Figure 10A and B. In Figure 10A, one can see that the front leg stays lifted, whereas the middle leg is on the ground, and the hind leg continues its normal stepping. An analogous result was obtained when the middle leg was kept lifted (Fig. 10C). In the case displayed in Figure 10B, the front leg did not stop but performed coordinated stepping with the hind leg, while the middle leg remained on the ground. Whether we saw the first or the second type occurring, depended on the timing of the decoupling command within a stepping period (compare the position of the arrows in Fig. 10A–C). Within one stepping period, the intervals of occurrence of both types were almost equally long, if the starting coordination pattern was tetrapod. With tripod as starting coordination pattern, coordinated stepping of the front and hind leg (Fig. 10B) appeared in a longer subinterval within a period than the case shown in Figure 10A.

Coordinated walking of the front and hind leg after amputation of the middle leg was indeed occasionally observed in the experiments (Graham 1977; Grabowska et al. 2012). It is again surprising that the front leg could be decoupled together with the middle leg. This certainly is a disruption of coordinated walking, especially when the middle leg was kept lifted (Fig. 10C), since then two ipsilateral legs were permanently lifted.

Looking at all of the results of decoupling attempts of the middle leg, it is not surprising that the hind leg remains unaffected (in the model) in all of the above cases. The changes made to the intersegmental synapse or to the LD CPG neurons, or to the premotor INs of the middle leg could not have had any effect, whatsoever on the hind leg's CPGs. But we were faced with a more important problem: how to compare the simulation results with experimental data obtained after the amputation of the middle leg? To try to resolve this problem, we note that in this set of simulations, we only changed a small specific part of the intersegmental neuromuscular system that brings about coordinated stepping of the three ipsilateral legs. An amputation is a much more drastic interference with this system that destroys many of its important parts, e.g., muscles, sensory organs and pathways, and, almost certainly, the intraleg coordination of stepping, as well. Our results thus show the effect of the relatively small functional faults (damages), only, and not that of a complete leg amputation.

## Discussion

In the study reported here, we set out to investigate possible ways of decoupling a leg from the rest of the locomotor system during walking, using the 3‐leg model of a stick insect we had constructed (Tóth and Daun‐Gruhn 2016). Walking implies the coordinated stepping movements of the legs, in the insect of all six ones but because of constraints of the model, we had to restrict our study to the three ipsilateral legs. The physiological significance of these investigations has been that reversible or irreversible decoupling of a leg or pair of legs does occur or can be induced in insects. Irreversible decoupling is, most often, some kind of amputation of, or permanent damage to the leg(s) in question. Natural reversible decoupling of the front legs happen, for example, when the animal lifts its front legs in order to carry out search movements with them (e.g. Grabowska et al. 2012). In both cases, it is important to learn what kind of physiological mechanisms underlie the decoupling of legs. At present, we know little of these mechanisms. There is only sparse experimental material on this topic (e.g. Dürr 2001; Berg et al. 2011; Grabowska et al. 2012).

It seemed therefore especially apt to use an appropriate model for the study of putative mechanisms that can bring about (reversible) decoupling of legs. Thus, we resorted to our 3‐leg model (Tóth and Daun‐Gruhn 2016), which can successfully reproduce the most common coordination patterns stick insects exhibit, such as tripod and tetrapod, and the transition between them (Graham 1972; Grabowska et al. 2012). By means of this model, we could identify three specific ways by which decoupling of a specific leg (in the model) could be implemented. These are: (1) permanently changing the nature (excitatory, inhibitory) and the conductance of the intersegmental synaptic connections; (2) manipulating the input to the CPG of the LD system in a given (pro‐, meso‐ or metathoracic) segment; and (3) inhibiting or disinhibiting the appropriate MNs via their premotor inhibitory INs (Fig. 2). We carried out simulations to test these possibilities for all three legs in the model. We did this for both tetrapod and tripod starting coordination pattern prior to decoupling. After decoupling, the model mimicked tetrapod coordination pattern in both cases, as observed in most of the experiments (Graham 1977; Grabowska et al. 2012), with the constraint of just looking at the ipsilateral legs. By inclusion of both starting conditions, we wanted to keep the possibility of comparing the stepping behavior of young (first instar) animals that most often use tripod coordination pattern (Graham 1977; D. Wetzel and J. Egert, personal communication) beside that of adult animals for which tetrapod coordination pattern is characteristic (Graham 1972; Grabowska et al. 2012).

We found that decoupling of the front leg could be achieved by permanently lifting the front leg which automatically put it in a protracted and stretched state (see Results). This lifted state of the front leg seems to make sense from the physiological point of view, since the stick insect often uses its front legs for search movements in the air (Berg et al. 2011; Grabowska et al. 2012). It should therefore be able to change the function of the front legs from stepping to searching, and the other way around, fast, with no long transition time from one state to the other. It appears that among the three ways found to do that in the model, the disinhibition of the premotor inhibitory IN to the depressor MN and the simultaneous inhibition of the premotor inhibitory IN to the levator MN of the LD system can do that most reliably and efficiently (Fig. 5). In this case, the activity of the MNs, hence the corresponding muscles, is fully determined by the activity of the premotor INs. The input to the CPG of the LD system is not affected, nor is the function of the intersegmental coordinating synapses. The coordinated stepping (tetrapod or tripod coordination pattern) can therefore resume immediately once the decoupling ends, that is, once the premotor INs receive again their ‘normal’ input. This process is independent of the starting coordination pattern.

Decoupling via the premotor inhibitory INs of the LD system worked well for the hind leg, too. In this case, however, the levator premotor IN (neuron IN30 in Fig. 1D) underwent complete disinhibition (zero input), and the depressor premotor IN (neuron IN29 in Fig. 1D) was inhibited. As a result, the hind leg remained on the ground, retracted, and stretched (Fig. 7), which was observed in the experiments (Graham 1977; in adult animals: M. Grabowska and E. Godlewska, personal communication; in 1st and 2nd instar animals: D. Wetzel and J. Egert, personal communication). The decoupling was reversible in the latter but not in former experiments (Graham 1977), while it was always reversible in the simulations. Again, this result was independent of the starting coordination pattern. (Note that the hind leg remained stretched because the phase shift between the movement of the tibia of the hind leg and the movement of the two other legs is half of a stepping period (see Fig. 3A, for example).

Using the same type of decoupling for the middle leg led also to fixed positions of that leg: it remained either on the ground (retracted, flexed), or stayed in the complementary position (lifted, protracted, stretched) depending on the input to the corresponding premotor INs of the LD system of the middle leg. However, the behavior of the other legs was different from that in the previous cases (see below).

As far as the other methods of decoupling are concerned, the first one (change of the intersegmental synapse) proved effective for the front leg but failed to switch off the (periodic) movements of the middle leg (Figs 8, 9). The attempts to decouple a leg by changing the input to the CPG of the LD system of the leg to be decoupled yielded mixed results. It worked for the front leg if the excitatory synaptic connection on the levator CPG neuron (C3 in Fig. 1D) and the inhibitory one on the depressor CPG neuron (C4 in Fig. 1D) were strong enough. But even these conditions did not always suffice. The decoupling took place only, if the decoupling command was evoked in a certain phase range of a stepping period (Fig 4A). This range was virtually negligible with tripod starting coordination pattern, and about half of a stepping period with tetrapod starting coordination pattern. When the decoupling failed, the three legs simply continued their coordinated stepping using tetrapod coordination pattern (Fig. 4B). In the case of the hind leg, the phase range of successful decoupling dominated the stepping period (Fig. 6A) but failed ones were still clearly discernible: the hind leg being permanently lifted in a protracted and flexed state (Fig. 6B). For the middle leg, decoupling by changing the input to the CPG of the LD system of the hind leg completely failed (Fig. 9). Somewhat surprisingly, however, a perfect decoupling of the front leg could be achieved with starting tetrapod coordination pattern. When combining the decoupling methods (1) and (2), method (1) (changing the intersegmental synapse) always dominated method (2) (changing the input to the CPG of the LD system).

In summary, the most efficient and reliable way of decoupling of either leg is the appropriate inhibition and disinhibition of the premotor INs of the MNs of the LD system of the leg to be decoupled.

A very important point that had to be examined in the simulations was: what happens to the remaining two legs? Do they continue performing co‐ordinated stepping or is the coordination disrupted? Is their behavior in agreement with the experimental data? These questions concern properties that can easily be checked in the experimental and the simulation results. We found that decoupling of the front leg preserved the coordinated stepping of the middle and hind leg whatever method of decoupling was used. This seems, to some extent, obvious, since rhythmic stepping activity propagates from the hind leg to the front leg both in the stick insect and in the model. That is, the hind leg is the “source” of rhythmicity. On the other hand, the effects of decoupling the hind leg would therefore not have been foreseen so easily because of this property of the hind leg's LD local control network (Tóth and Daun‐Gruhn 2016). Nevertheless, decoupling of the hind leg did not abolish the coordinated stepping of the front and middle leg. Just like in the experiments (Graham 1977; Grabowska et al. 2012), the coordinated stepping of these legs, using tetrapod as starting coordination pattern, continued after decoupling of the hind leg. This shows that in the model, and probably in the animal, too, the LD local control network of the middle leg could take over the role of the “source” of the rhythmic stepping activity upon decoupling of the hind leg without any external intervention (other than the decoupling itself). This agreement between experiment and simulation can also be taken as a positive test result for the validity of the model.

Finally, decoupling of the middle leg did provide a somewhat mixed and complex picture. The result of the simulation depended heavily on the phase of the stepping period in which the decoupling command was evoked. In addition, the results obtained with starting tripod and tetrapod coordination patterns also differed occasionally. In some cases, the front and hind leg continued coordinated stepping even though the spatial position of the middle leg was completely fixed (by the activity of the premotor INs). There were, however, cases in which the front leg was lifted, hence no coordinated stepping of the front and hind leg took place. In the experiments, coordinated stepping (in tetrapod) was occasionally observed in the animals (Graham 1977; Grabowska et al. 2012), especially if they used tripod coordination pattern prior to decoupling (amputation or restraint). In our simulations, too, coordinated stepping of the front and hind leg occurred more often with tripod starting coordination pattern. Since, in the majority of the experiments, the middle leg was amputated, that is, irreversibly decoupled, the conditions in the experiments and the simulations were not completely analogous. To mimic conditions after amputation, we ought to have disabled even more functions of the model, such as intraleg coordination, not just the intersegmental synapses, the CPG neurons and the premotor inhibitory INs of the LD system. This would, most likely, have led to more failures of coordinated walking in the model. We did not carry out such simulations in order to be able to concentrate on the examination of the crucial parts of the model that are responsible for producing coordinated movements. These are clearly the CPGs of the LD control network, the corresponding premotor INs in these networks, and the intersegmental synaptic pathways converging on the CPG neurons of the LD system of the front and middle leg. Had we extended the set of functional units to be disabled, this could easily have led to a situation in which it would have been quite difficult to find out what changes would have what effects in the model. We think that our restriction to the three aforementioned functional units of decoupling has therefore been reasonable both from the methodological and the physiological point of view.

We consider the relevance and, perhaps, the significance of this study to be twofold. First, the model could reproduce the types of special behavior that the stick insect exhibits if some of its legs are reversibly or irreversibly decoupled from the rest of the locomotor system. This is an important positive test result with the model, that underpins its relevance in studying legged locomotion of the stick insect, and perhaps, of other insect species. Second, the model offers “solutions” for putative decoupling mechanisms that may be at work in the animal. The fact that, in some cases, more than one solution was found may be interpreted as a sign of existing redundancy in the animal, which is of paramount importance in all living organisms. Indeed, stick insects possess both segmental CPGs and premotor neuronal networks that are much more complex than those in the model. They must also have intersegmental synaptic pathways that participate in the intersegmental coordination of leg movements. Our model, by exploring various possible decoupling mechanisms, provides a choice of the possible ways of intersegmental organization, hence a basis for further experiments.

## Conflict of Interest

None declared
